# Adipsin Serum Concentrations and Adipose Tissue Expression in People with Obesity and Type 2 Diabetes

**DOI:** 10.3390/ijms23042222

**Published:** 2022-02-17

**Authors:** Margarete Milek, Yusef Moulla, Matthias Kern, Christine Stroh, Arne Dietrich, Michael R Schön, Daniel Gärtner, Tobias Lohmann, Miriam Dressler, Peter Kovacs, Michael Stumvoll, Matthias Blüher, Esther Guiu-Jurado

**Affiliations:** 1 Medical Department III—Endocrinology, Nephrology, Rheumatology, University of Leipzig Medical Center, 04103 Leipzig, Germany; margarete.milek@me.com (M.M.); peter.kovacs@medizin.uni-leipzig.de (P.K.); michael.stumvoll@medizin.uni-leipzig.de (M.S.); 2 Clinic for Visceral, Transplantation and Thorax and Vascular Surgery, University Hospital Leipzig, 04103 Leipzig, Germany; yusef.moulla@medizin.uni-leipzig.de (Y.M.); arne.dietrich@medizin.uni-leipzig.de (A.D.); 3 Helmholtz Institute for Metabolic, Obesity and Vascular Research (HI-MAG) of the Helmholtz Zentrum München at the University of Leipzig and University Hospital Leipzig, 04103 Leipzig, Germany; matthias.kern@helmholtz-muenchen.de; 4 Department of General, Abdominal and Pediatric Surgery, Municipal Hospital, 07548 Gera, Germany; christine.stroh@srh.de; 5 Städtisches Klinikum Karlsruhe, Clinic of Visceral Surgery, 76133 Karlsruhe, Germany; m.schoen@klinikum-karlsruhe.de (M.R.S.); gaertner@klinikum-karlsruhe.de (D.G.); 6 Municipal Clinic Dresden-Neustadt, 01129 Dresden, Germany; tobias.lohmann@klinikum-dresden.de (T.L.); miriam.dressler@klinikum-dresden.de (M.D.); 7 Deutsches Zentrum für Diabetesforschung e.V., 85764 Oberschleißheim, Germany

**Keywords:** adipose tissue, adipsin, obesity, T2D

## Abstract

(1) Adipsin is an adipokine that may link increased fat mass and adipose tissue dysfunction to obesity-related cardiometabolic diseases. Here, we investigated whether adipsin serum concentrations and adipose tissue (AT) *adipsin* mRNA expression are related to parameters of AT function, obesity and type 2 diabetes (T2D). (2) Methods: A cohort of 637 individuals with a wide range of age and body weight (Age: 18–85 years; BMI: 19–70 kg/m^2^) with (n = 237) or without (n = 400) T2D was analyzed for serum adipsin concentrations by ELISA and visceral (VAT) and subcutaneous (SAT) *adipsin* mRNA expression by RT-PCR. (3) Results: Adipsin serum concentrations were significantly higher in patients with T2D compared to normoglycemic individuals. We found significant positive univariate relationships of adipsin serum concentrations with age (r = 0.282, *p* < 0.001), body weight (r = 0.264, *p* < 0.001), fasting plasma glucose (r = 0.136, *p* = 0.006) and leptin serum concentrations (r = 0.362, *p* < 0.001). Neither VAT nor SAT *adipsin* mRNA expression correlated with adipsin serum concentrations after adjusting for age, sex and BMI. Independent of T2D status, we found significantly higher *adipsin* expression in SAT compared to VAT (4) Conclusions: Our data suggest that adipsin serum concentrations are strongly related to obesity and age. However, neither circulating adipsin nor *adipsin* AT expression reflects parameters of impaired glucose or lipid metabolism in patients with obesity with or without T2D.

## 1. Introduction

Obesity can be considered a slow motion pandemic, as its prevalence has been tripled worldwide since 1975 [1,2,3]. Obesity increases the risk of cardiovascular, metabolic and multiple other comorbidities [4,5], but the individual risk for these diseases may vary and is at least partly related to distinct alterations in adipose tissue (AT), including its endocrine function [6,7]. In genetically susceptible people, increased energy intake results in AT accumulation and is often accompanied by adipocyte hypertrophy [8,9], AT inflammation and heterogeneous body fat distribution [6,10]. Altered secretion of adipokines and changes in AT metabolites release may link obesity to AT dysfunction and obesity-related cardiometabolic diseases [4,6,7,11,12]. In the past two decades, hundreds of adipokines have been discovered [13]. For many of these adipokines, we only have an incomplete understanding about their mechanism of action, regulation of expression and clinical relevance [14]. 

Adipsin is an adipokine that represents one of the major proteins expressed by adipocytes [15]. First reported in 1987, adipsin has been identified as complement factor D, an integral part of the complement system [16,17,18], which induces amplification of the alternative complement pathway [18,19]. In this process, adipsin is integrated into an enzymatic cascade that releases the C5–C9 membrane attack complex and anaphylatoxins like C3a and C5a [20]. Adipsin is predominantly synthesized by AT cells [17] and associations between circulating adipsin and parameters of obesity and glucose metabolism have been found recently [21,22,23]. Adipsin catalyzes the release of complement factor C3a, which has been shown to stimulate insulin production in pancreatic ß-cells [20]. Adipsin serum concentrations are reduced in patients with type 2 diabetes (T2D) and ß-cell failure [20]. Furthermore, adipsin facilitates glucose uptake and increases triglyceride synthesis in adipocytes [24]. Mice with a genetic ablation of adipsin are characterized by impaired glucose homeostasis in response to diet-induced obesity [18,20]. The model revealed an important role of adipsin in the regulation of normal insulin secretion. Taken together, these recent findings propose adipsin as an important AT-secreted factor that may link obesity and adipocyte dysfunction to impaired β-cell function and cardiometabolic diseases.

We therefore tested the hypothesis that *adipsin* gene expression in human AT and its serum concentrations are related to obesity, fat distribution and parameters of AT function and glucose metabolism. We sought to determine whether AT *adipsin* mRNA is differentially expressed in subcutaneous and visceral fat depots and whether it correlates to adipsin serum concentrations in patients with obesity with or without T2D.

## 2. Results

### 2.1. Adipsin mRNA Is Higher in SAT Compared to VAT but Not Related to Obesity and T2D 

**Figure 1:**
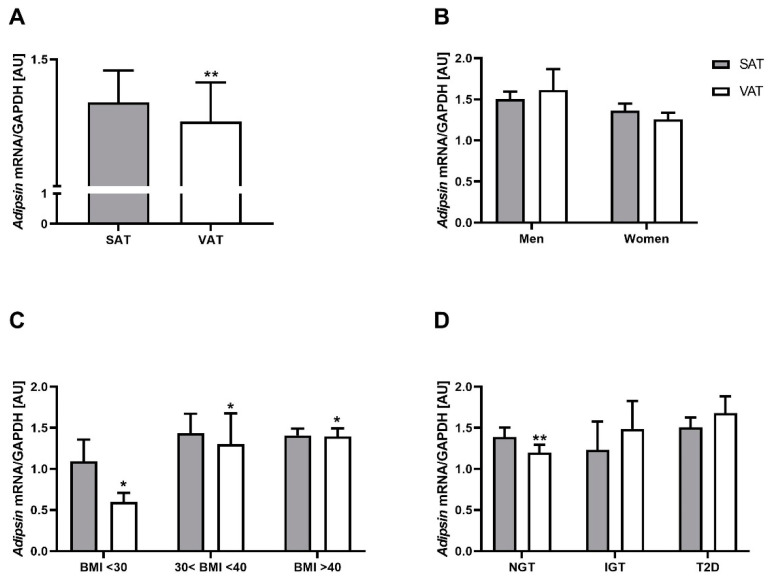
*Adipsin* mRNA expression in visceral (VAT) and subcutaneous (SAT) adipose tissue in control individuals and patients with obesity and/or type 2 diabetes (T2D). *Adipsin* gene expression in (**A**) the entire study population (n = 607); (**B**) men (n = 163) and women (n = 444); (**C**) subgroups of controls (BMI < 30 kg/m^2^, n = 21), patients with moderate obesity (30 kg/m^2^ < BMI < 40 kg/m^2^, n = 48) or morbid obesity (BMI > 40 kg/m^2^, n = 538); (**D**) subjects with normal glucose tolerance (NGT, n = 274), impaired glucose tolerance (IGT, n = 14) or T2D (n = 232). Statistical significance at * *p* < 0.05 and ** *p* < 0.01 when comparing *adipsin* mRNA expression between both adipose tissues. Data are given as means ± SEM.

Analysis of 607 paired AT samples showed significantly higher *adipsin* mRNA expression in SAT compared to VAT (Figure 1A), regardless of the degree of obesity or T2D status (Figure 1). The results showed a trend towards increased expression of VAT *adipsin* mRNA according to the degree of obesity (Figure 1C) and to the diabetes status (Figure 1D). There was no significant difference between women and men in *adipsin* gene expression in both fat depots (Figure 1B).

We performed additional analyses in subgroups of individuals with a BMI < 30 kg/m^2^ (n = 21), individuals with a BMI between 30 and 40 kg/m^2^ (n = 48) and patients with a BMI > 40 kg/m^2^ (n = 538). SAT and VAT *adipsin* mRNA expressions were not significantly different between these BMI subgroups (Figure 1C). 

**Figure 2:**
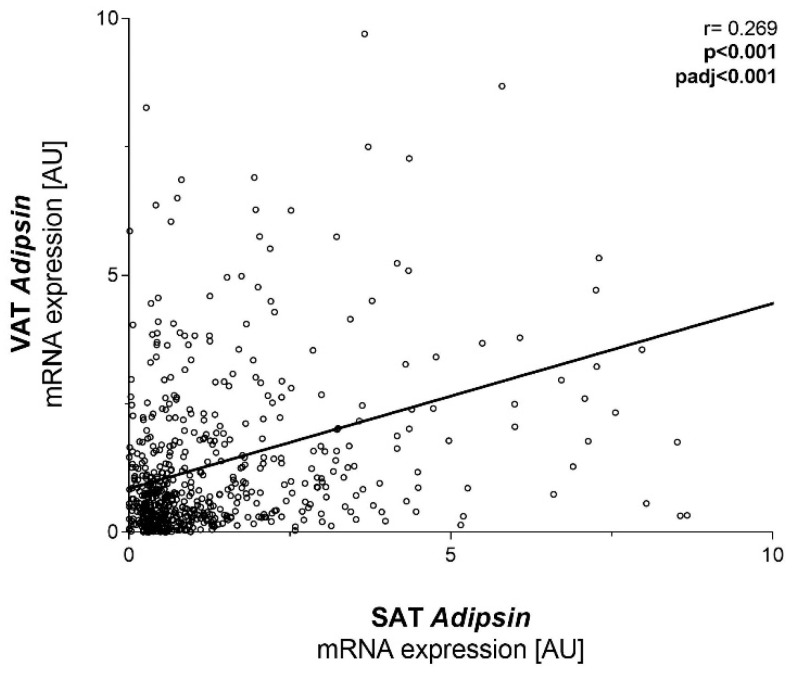
Linear regression of visceral and subcutaneous *adipsin* mRNA expression. Adjusted *p*-values were calculated in linear regression after adjusting for age, sex and BMI. VAT, visceral adipose tissue; SAT, subcutaneous adipose tissue.

**Figure 3:**
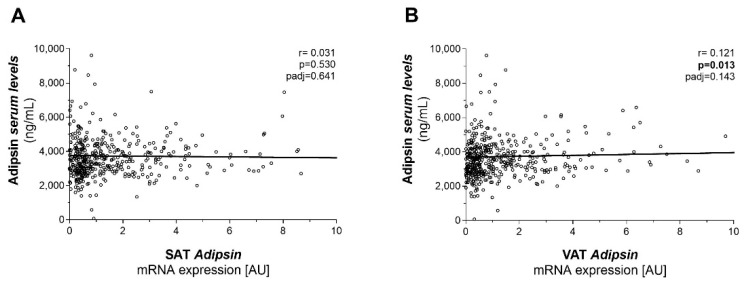
Linear regression of adipsin serum concentrations with (**A**) subcutaneous adipose tissue (SAT) and (**B**) visceral (VAT) *adipsin* mRNA expression. Adjusted *p*-values were calculated in linear regression after adjusting for age, sex and BMI.

We further stratified study participants according to glucose tolerance and T2D status. SAT and VAT *adipsin* mRNA expressions were not significantly different across participants with normal or impaired glucose tolerance and T2D (Figure 1D). Linear regression analysis revealed a significant positive relationship between SAT and VAT *adipsin* mRNA expression, even after adjustment for BMI, sex and age (Figure 2). SAT *adipsin* mRNA expression significantly correlated with the waist-to-hip ratio (WHR) and remained significant even after respective adjustment. VAT *adipsin* mRNA expression significantly correlated with serum leptin concentrations adjusted for age, sex and BMI (Table 1). VAT *adipsin* mRNA expression significantly correlated with circulating adipsin levels in the univariate regression analysis, but this association did not withstand adjustment for age, sex and BMI (Figure 3). Moreover, we did not observe a significant association between SAT *adipsin* expression and serum adipsin concentrations (Figure 3).

### 2.2. Adipsin Serum Concentrations Are Higher in Patients with Obesity and T2D

**Figure 4:**
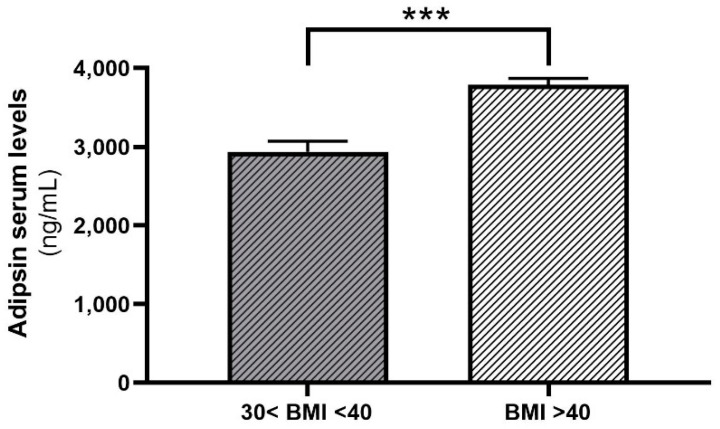
Adipsin serum concentrations in subgroups of moderately obese (30 kg/m^2^ < BMI < 40 kg/m^2^, n = 38) and morbidly obese patients (BMI > 40 kg/m^2^, n = 414). Statistical significance at *** *p* < 0.001. Data are given as means ± SEM.

Adipsin serum concentrations were not different between women and men (*p* = 0.549). Patients with a BMI > 40 kg/m^2^ had significantly higher adipsin serum concentrations compared to patients in the BMI range between 30 and 40 kg/m^2^ (Figure 4). Importantly, we found significant positive relationships between adipsin serum concentrations, body weight, BMI and leptin serum concentrations (Table 1). 

**Figure 5:**
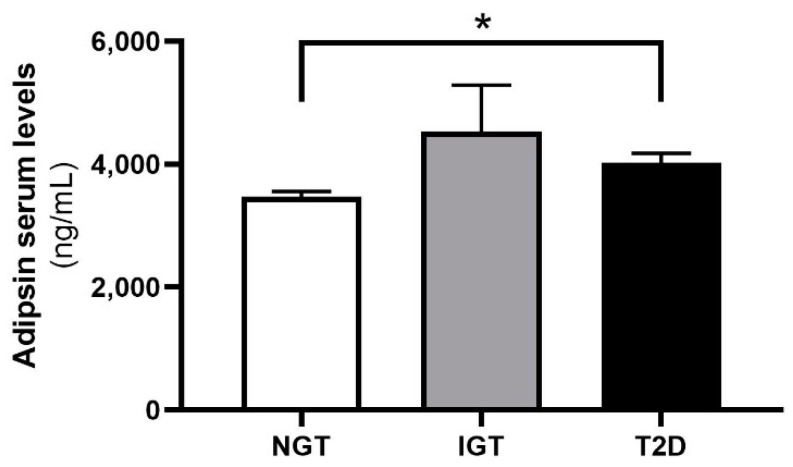
Adipsin serum concentrations in subgroups of subjects with normal glucose tolerance (NGT, n = 197), impaired glucose tolerance (IGT, n = 15) or T2D (n = 168). Statistical significance at * *p* < 0.05. Data are given as means ± SEM.

Adipsin serum concentration was significantly higher in patients with T2D compared to normoglycemic controls (Figure 5). There was no significant difference in circulating adipsin levels between patients with T2D and impaired glucose tolerance (Figure 5). Furthermore, we stratified patients with T2D for whom we had information on their diabetic complications, such as nephropathy and retinopathy, among others, according to the presence of one or more diabetic complications (Subjects with T2D but without diabetic complications, n = 75; subjects with T2D and one or more diabetic complications, n = 57). However, adipsin serum concentrations were not statistically different between groups of T2D patients with or without diabetic complications (*p* = 0.965). Furthermore, adipsin serum levels significantly correlated with age and fasting plasma glucose (FPG) (Table 1). However, the correlation of serum adipsin with FPG did not remain significant after adjustment for sex, age and BMI (Table 1). 

## 3. Discussion

Adipsin has been suggested to play a role in the development of obesity and its comorbidities [17,21,22,23]. Circulating adipsin has been shown to decline in several animal models for obesity and diabetes [25]. Moreover, rodent studies demonstrated that adipsin treatment has beneficial effects on insulin secretion and glucose parameters [20,21]. Circulating adipsin has been proposed to predict ß-cell failure in a subgroup of patients with T2D [20]. On the other hand, human studies found positive correlations between adipsin serum concentrations and BMI [21,26]. Interestingly, increased adipsin serum concentrations seem to be associated with acquired syndromic partial lipodystrophy [27]. 

A mechanistic role of adipsin in the development of obesity and T2D could be related to its function in regulating factors of the complement system, most importantly C3 [28,29,30]. C3a, which is part of the complement system, is released by the upstream catalytic action of adipsin and may then stimulate insulin synthesis in pancreatic ß-cells under hyperglycemic conditions [20]. Indeed, deficiency or antagonism of the C3a receptor 1 protects mice against obesity, reduces AT inflammation and improves systemic insulin sensitivity [29,30]. In this context, obesity-associated AT inflammation could be aggravated by complement activation and subsequent infiltration of immune cells into AT [31]. Consequently, adipsin may play a role in the pathogenesis of human obesity and its comorbidities including T2D. We therefore tested the hypothesis that AT *adipsin* mRNA expression and adipsin serum concentrations are related to parameters of obesity, glucose metabolism and AT distribution. 

In the context of a cross-sectional study including 637 individuals with a wide range of body weight and metabolic parameters, we analyzed relationships between adipsin serum concentrations and/or *adipsin* mRNA expression in visceral and subcutaneous AT with body weight, anthropometric and metabolic traits. Our approach extends previous human adipsin studies [15,17,20,21,22] by providing parallel data on circulating adipsin and *adipsin* gene expression from two abdominal fat depots.

As main findings, our analyses revealed that adipsin serum concentrations were significantly higher in patients with T2D compared to normoglycemic individuals and that adipsin serum concentrations significantly correlated with age, body weight, BMI, fasting plasma glucose and leptin serum concentrations. AT *adipsin* mRNA expression did not correlate with adipsin serum concentrations after adjustment for age, sex and BMI. Independent of T2D status, we found significantly higher *adipsin* expression in SAT compared to VAT. The observed fat depot differences in *adipsin* expression are in accordance with data from the Genotype-Tissue Expression (GTEx) repository [32] and from a study of 16 men and 16 women with a BMI between 20 and 54 kg/m^2^ [33]. Given that VAT and SAT *adipsin* gene expressions are significantly correlated, fat-depot specific differences in the regulation of *adipsin* expression may be related to intrinsic differences between these depots, including cellular composition rather than a depot-specific regulation of expression. Our findings indicate that adipsin may not be exclusively produced by AT and that, to some extent, it is released from other tissues. GTEx data show increased *adipsin* expression levels, of course, in SAT and VAT, but also in coronary arteries, tibial nerve and the female breast and vagina in human subjects [32]. Further studies are required to define the contribution of tissues other than AT to circulating adipsin levels. 

Higher *adipsin* gene expression in SAT may reflect lower AT stressors and lower AT immune cell infiltration compared to visceral fat depots [34,35,36] suggesting that adipsin may predict “healthier AT”. In accordance with another study [33], we found a trend towards increased expressions of VAT *adipsin* mRNA with increasing BMI and T2D. It has been suggested that adipsin may reflect obesity subphenotypes, including metabolically healthy obesity [20]. On the other hand, SAT *adipsin* gene expression significantly negatively correlated with WHR, suggesting that *adipsin* expression either reflects fat distribution or may contribute to the regulation of regional body fat accumulation. Supporting this hypothesis and recent data from other groups [37,38,39], we also found that patients with obesity and T2D have significantly higher circulating adipsin compared to normoglycemic individuals in the same BMI range. However, these differences were not reflected in differences in *adipsin* AT expression among patients with obesity discordant for the T2D status. Studies that excluded patients with morbid obesity (including only BMI < 40 kg/m^2^) did not find differences in circulating adipsin between patients with T2D and controls [40,41].

We found a positive correlation between adipsin serum concentrations and VAT *adipsin* expression. However, this correlation did not remain significant after adjustment for potential confounding factors age, sex and BMI. Although visceral fat depot is characterized by lower *adipsin* expression compared to SAT, it could contribute to higher adipsin serum concentrations observed in patients with T2D, thereby further linking visceral fat distribution to metabolic alterations of obesity. Our study design did not allow drawing conclusions about a mechanistic role of adipsin in the link between obesity, VAT dysfunction and the development of impaired glucose metabolism. Future studies are needed to gain further knowledge about the role of adipsin in the development of obesity and T2D. 

Linear regression analyses further revealed a positive relationship between serum adipsin levels and age, which remained significant after adjustment for sex and BMI. Our results are in contrast to data from a Chinese study that did not find associations between age and circulating adipsin [40]. However, we confirm data from other studies [37,42,43] that adipsin serum levels are not significantly different between men and women. In addition, our results showed that *adipsin* in SAT and VAT was not differently expressed in women and men. 

Our data confirm previous studies in smaller cohorts [21,25,26,44,45,46] that adipsin serum concentration positively correlates with body weight and BMI. The association between adipsin and BMI has been attributed to effects of increased fat mass [45], however, univariate regression analyses did not identify body fat mass as a significant correlate of circulating adipsin. On the other hand, leptin—another correlate of body fat mass—positively correlated with adipsin, suggesting that our analyses of body fat associations may lack statistical power due to a lower number of individuals for whom body composition measurements were available. 

Our results are also consistent with the assumptions by Lo et al., who found increased adipsin levels in the early stages of the metabolic syndrome, which are attributed to the increased amount of AT in obesity to compensate for the decreased adipsin synthesis per AT unit [20]. Furthermore, we found a significantly positive relationship between adipsin serum levels and fasting plasma glucose, at least in unadjusted univariate correlation analyses. The fact that statistical significance of the relationship between adipsin and FPG was lost after adjusting for age and BMI suggests that these confounding factors are stronger determinants of adipsin serum concentrations. Previously reported inverse correlations between adipsin and FPG [21,40,41] may be explained by differences between these cohorts and ours with regard to the duration of hyperglycemia and other indices of the metabolic status [20]. Lo et al. suggest that higher adipsin synthesis reflects early stages of T2D and plays a compensatory role in the organisms attempt to normalize glucose and lipid metabolism [20]. During T2D progression, adipsin levels might decrease in the context of AT dysfunction and, eventually, ß-cell failure may develop [20]. In this context, Lo et al. found decreased circulating adipsin levels in patients with T2D and ß-cell failure compared to patients with T2D and preserved ß-cell function [20]. Our findings do not support previous reports suggesting that adipsin serum concentrations are lower in animal models for diabetes and people with T2D [20,21,40,47,48]. Low circulating adipsin seems to be particularly associated with ß-cell failure in patients with T2D [20,21,40]. We found higher adipsin serum concentrations in patients with impaired glucose tolerance (IGT), a prediabetic state. These data suggest that patients with T2D included into our analyses may be characterized by a preserved or at least better ß-cell function compared to the patients with T2D included into previous studies [20,21,40]. Indeed, higher adipsin serum concentrations in people with IGT support the hypothesis that increased circulating adipsin may reflect an intrinsic mechanism of the organism to compensate for impaired insulin secretion. Longitudinal studies over the entire range from prediabetes to advanced T2D stages are required to test this hypothesis in the future. Taken altogether, adipsin might become clinically relevant as a future target to improve ß-cell function in patients with T2D. The manipulation of adipsin as a molecular switch to improve insulin secretion has been suggested to further study in the context of treating ß-cell failure in T2D [20].

In our study, we included T2D patients with concomitant medication including metformin, DPP-4 and SGLT-2 inhibitors or GLP-1 receptor agonists. We can, therefore, not exclude that specific anti-diabetic medication may affect adipsin serum concentrations. In this context, Taşdemir et al. reported that diabetic rats treated with metformin have increased plasma adipsin levels compared to untreated diabetic rats [49]. Moreover, we investigated whether adipsin serum concentrations reflect T2D complications such as cardiovascular disease, retinopathy or nephropathy. In this context and against the hypothesis that circulating adipsin may decline with more advanced stages of T2D, we did not find differences in circulating adipsin between subgroups of T2D patients with or without secondary diabetes complications. 

Our study has some limitations. First, we predominantly included patients undergoing bariatric surgery with a BMI > 40 kg/m^2^. This high body weight bias needs to be acknowledged. Therefore, our results may not reflect associations between adipsin and metabolic traits in the lower BMI range. In addition, we cannot exclude effects of concomitant medications such as antidiabetic, antihypertensive medications, statins or pain killers, although there was no statistical evidence for an interference between adipsin measurements and specific pharmacotherapies. Although serum samples were taken immediately prior to surgery, the pre-operative fasting period may differentially influence *adipsin* AT expression and adipsin serum concentrations. Moreover, despite adipsin being released from AT and thereforeis considered an adipokine, its role as complement factor D and alternative pathway convertase cofactor in the cleavage of C3 is functionally relevant [16,18]. Therefore, data on complement levels such as total C3 levels would have further extended our general view on presented data. Since we are not able to provide data on complement factor serum concentrations, we have to acknowledge the lack of data on the complement factor status of our study participants as a limitation.

In conclusion, our data suggest that adipsin serum concentrations are strongly related to obesity and age. However, neither circulating adipsin nor *adipsin* AT expression reflects the parameters of impaired glucose or lipid metabolism in patients with obesity with or without T2D.

## 4. Materials and Methods

### 4.1. Subjects

We included 637 metabolically well-characterized participants of the Leipzig Obesity BioBank recruited at four bariatric surgery centers in Leipzig, Karlsruhe, Dresden and Gera (all in Germany) (Table 2). All subjects underwent clinical phenotyping as described previously [7,50,51]. All subjects had a stable weight, defined as no fluctuations of > 2% of body weight for at least 3 months before surgery. According to American Diabetes Association (ADA) criteria [52], 248 study participants (~39%) were diagnosed with T2D. We defined the following exclusion criteria: (i) thyroid dysfunction, (ii) alcohol or drug abuse, (iii) pregnancy and (iv) treatment with thiazolidinediones. The study was approved by the ethics committee of the University of Leipzig (Approval numbers: 159-12-21052012 and 017-12-23012012). The study design follows the Declaration of Helsinki and all participants gave written informed consent prior to participation.

### 4.2. Measurement of Adipsin Serum Concentrations

Adipsin serum concentrations were analyzed in duplicate using enzyme-linked immunosorbent assay (ELISA) according to the manufacturer’s instructions (Quantikine^®^ ELISA Human Complement Factor D, R&D Systems, Minneapolis, MN, USA) in 455 patients. The age from these subjects ranged from 18 to 76 years and body mass index (BMI) from 30 to 70 kg/m^2^. Adipsin assay sensitivity was 0.025 pg/mL and inter-assay and intra-assay coefficients of variation were less than 9% and 6.4%, respectively.

### 4.3. Adipsin mRNA Expression Analysis in AT

Paired samples of abdominal omental AT (visceral, VAT) and subcutaneous AT (SAT) were obtained from 607 Caucasian men (n = 163) and women (n = 444) (Table 2), who underwent open abdominal surgery as described previously [50,51]. The age ranged from 18 to 85 years and body mass index (BMI) from 19 to 70 kg/m^2^ (Table 2). AT was immediately frozen in liquid nitrogen and stored at −80 °C. RNA was extracted from AT by using the RNeasy Lipid Tissue Mini Kit (Qiagen, Hilden, Germany), and qPCR was performed as described elsewhere [53,54]. Real-time quantitative PCR was performed with the TaqMan Assay predesigned by Applied Biosystems (Foster City, CA, USA) for the detection of human *adipsin* (Hs00157263_m1) and *glyceraldehyde 3-phosphate dehydrogenase* (*GAPDH*) (Hs 02786624_g1) mRNA expression in AT. All reactions were carried out in 96-well plates using the QuantStudio (TM) 6 Flex System Fast Real-Time PCR system. *Adipsin* mRNA expression was calculated relative to *GAPDH* mRNA expression.

### 4.4. Statistical Analyses

Prior to statistical analysis, non-normally distributed parameters were logarithmically (ln) transformed to approximate a normal distribution. Results are expressed as mean ± SD (standard deviation). Multivariate linear relationships between *adipsin* mRNA expression and phenotypic traits were assessed by generalized linear regression models. Differences in *adipsin* mRNA expression between visceral and subcutaneous AT were assessed using the paired Student’s *t*-test or one-way ANOVA. Statistical analyses were performed using SPSS/PC+ for Windows statistical package (Version 25.0; SPSS, Chicago, IL, USA).

## Figures and Tables

**Table 1:** Correlation analyses of visceral and subcutaneous *adipsin* mRNA expression and adipsin serum levels with anthropometric and metabolic parameters (n = 637).

	SAT *Adipsin* mRNA r (*p*-Value; *p*-Value adj)	VAT *Adipsin* mRNA r (*p*-Value; *p*-Value adj)	Adipsin Serum Levels r (*p*-Value; *p*-Value adj)
Age (years)	0.078 (0.057; 0.064)	0.074 (0.071; **0.025**)	**0.282** (**<0.001**; **<0.001**)
Body weight (kg)	−0.018 (0.664; 0.219)	0.058 (0.165; 0.460)	**0.264** (**<0.001**; **<0.001**)
Height (m)	−0.058 (0.166; 0.137)	−0.017 (0.687; 0.623)	0.037 (0.444; 0.534)
BMI (kg/m^2^)	0.008 (0.849; 0.482)	0.077 (0.060; 0.170)	**0.359** (**<0.001**; **<0.001**)
Waist circumference (cm)	−0.234 (0.080; 0.576)	0.089 (0.504; 0.670)	0.137 (0.439; 0.398)
Hip circumference (cm)	0.039 (0.803; 0.971)	0.175 (0.262; 0.051)	0.099 (0.66; 0.45)
WHR	**−0.49** (**0.001**; **<0.001**)	0.03 (0.849; 0.666)	−0.108 (0.633; 0.170)
Body fat (%)	0.048 (0.569; 0.290)	0.081 (0.330; 0.561)	0.073 (0.423; 0.101)
FPG (mmol/L)	0.025 (0.576; 0.338)	0.022 (0.624; 0.748)	**0.136** (**0.006**; 0.948)
FPI (pmol/L)	−0.048 (0.588; 0.911)	−0.100 (0.256; 0.875)	0.126 (0.176; 0.53)
HbA1c (%)	0.018 (0.770; 0.272)	0.036 (0.547; 0.727)	0.044 (0.470; 0.095)
HOMA-IR	−0.074 (0.402; 0.777)	−0.100 (0.253; 0.574)	0.076 (0.410; 0.794)
Total Cholesterol (mmol/L)	0.003 (0.955; 1.00)	0.019 (0.751; 0.278)	−0.067 (0.326; 0.896)
HDL-C (mmol/L)	0.042 (0.476; 0.462)	−0.050 (0.401; 0.615)	−0.012 (0.858; 0.486)
LDL-C (mmol/L)	0.012 (0.842; 0.907)	−0.025 (0.681; 0.455)	0.007 (0.919; 0.508)
Triglycerides (mmol/L)	0.018 (0.753; 0.671)	0.033 (0.571; 0.753)	−0.068 (0.313; 0.694)
CrP (mg/L)	−0.065 (0.115; 0.158)	−0.049 (0.238; 0.297)	0.039 (0.411; 0.825)
Leptin serum levels (ng/mL)	0.008 (0.875; 0.44)	**0.135** (**0.001**; **0.021**)	**0.362** (**<0.001**; **<0.001**)

BMI, body max index; FPG, fasting plasma glucose; FPI, fasting plasma insulin; HDL-C, high density lipoprotein cholesterol; LDL-C, low density lipoprotein cholesterol; r, Pearson correlation coefficient; SAT, subcutaneous adipose tissue; TG, triglycerides; VAT, visceral adipose tissue; WHR, waist to hip ratio. Non-normally distributed parameters were logarithmically transformed to approximate a normal distribution. *p*-values adj were calculated in linear regression after adjusting for age, sex and BMI, except for weight and % body fat, which were adjusted only for age and sex. Significant correlations (*p* < 0.05) are highlighted in bold.

**Table 2:** Anthropometric and metabolic characterization of the cohort.

	BMI < 30 kg/m^2^ (n = 21)	BMI 30–40 kg/m^2^ (n = 52)	BMI > 40 kg/m^2^ (n = 564)
Age (years)	66.13 ± 10.56	48.41 ± 11.34 ***	46.60 ± 11.93 ***
Men/Women (n)	14/7	14/38	148/416
T2D (n)	4	22	211
Body weight (kg)	75.90 ± 12.28	107.41 ± 14.19 ***	142.81 ± 26.36 ***^,###^
Height (m)	1.74 ± 0.97	1.70 ± 0.084	1.70 ± 0.098
BMI (kg/m^2^)	25.05 ± 2.33	36.70 ± 2.84 ***	49.52 ± 7.21 ***^,###^
Body fat (%)	22.91 ± 5.01	42.97 ± 9.40 ***	48.42 ± 10.09 ***
Waist circumference (cm)	96.39 ± 14.95	122.50 ± 9.20 *	143.667 ± 14.22 ***
Hip circumference (cm)	97.11 ± 10.64	124 ± 11.32 *	149.42 ± 14.50 ***^,#^
WHR	0.99 ± 0.11	0.99 ± 0.17	0.99 ± 0.09
FPG (mmol/L)	5.72 ± 0.66	6.12 ± 1.96	6.40 ± 2.43
FPI (pmol/L)	47.33 ± 29.99	115.78 ± 88.57	145.47 ± 104.87
HbA1c (%)	5.73 ± 0.45	6.25 ± 1.46	5.99 ± 1.139
HOMA-Index	1.67 ± 1,08	5.00 ± 4.16	5.90 ± 6.00
Total Cholesterol (mmol/L)	5.40 ± 1.25	5.30 ± 1.27	5.99 ± 1.139
HDL-Cholesterol (mmol/L)	1.25 ± 0.25	1.26 ± 0.30	1.16 ± 0.61
LDL-Cholesterol (mmol/L)	3.31 ± 0.98	3.46 ± 1.15	3.09 ± 0.93
Triglycerides (mmol/L)	1.26 ± 0.57	1.98 ± 1.22	2.06 ± 2.24
CrP (mg/L)	9.75 ± 11.77	7.00 ± 10.48	12.57 ± 17.58
AT *adipsin* mRNA (n)	21	48	538
Adipsin serum levels (n)	4	38	414
Parallel *adipsin* mRNA and serum levels data (n)	4	32	388

Data are given as means ± SD. Statistical significance at *** *p* < 0.001 and at * *p* < 0.05 when compared with BMI < 30 kg/m^2^ group. Statistical significance at ^###^ *p* < 0.001 and at ^#^ *p* < 0.05 when compared with BMI 30–40 kg/m^2^ group. AT, adipose tissue; BMI, body max index; FPG, fasting plasma glucose; FPI, fasting plasma insulin; HDL-C, high density lipoprotein cholesterol; LDL-C, low density lipoprotein cholesterol; SAT, subcutaneous adipose tissue; VAT, visceral adipose tissue; WHR, waist to hip ratio.

## Data Availability

Not applicable.
